# Two new species of the genus *Meleonoma* Meyrick from China (Lepidoptera, Gelechioidea, Xyloryctidae)

**DOI:** 10.3897/zookeys.871.35738

**Published:** 2019-08-12

**Authors:** Aihui Yin, Yanpeng Cai

**Affiliations:** 1 Morphological Laboratory, Guizhou University of traditional Chinese medicine, Guiyang 550025, Guizhou, China Guizhou University of traditional Chinese medicine Guiyang China

**Keywords:** Checklist, morphology, moth, taxonomy

## Abstract

Two new species of *Meleonoma* Meyrick, 1914a (Gelechioidea, Xyloryctidae) from southeastern China are described: *Meleonoma foliiformis* Yin, **sp. nov.** from Guangxi Province and *M. projecta* Yin, **sp. nov.** from Fujian Province. Adults and male genitalia are described in detail. A list of the *Meleonoma* species occurring in China is given. The taxonomic position of *Meleonoma* has been unstable, and under debate. Nonetheless, it is here tentatively placed in the family Xyloryctidae, following the latest molecular phylogenetic study concerning this genus.

## Introduction

The genus *Meleonoma* Meyrick was established in 1914 in the family Oecophoridae, for three species, with *Cryptolechia stomota* Meyrick, 1910a as the type species. Prior to this study, 35 valid species had been reported over the world, of which 14 had been discovered in China. The first ever described species of the genus was collected in Heilongjiang Province of China and published by Christoph under the original name of *Euteles flavimaculata* Christoph, 1882. However, it was not transferred into *Meleonoma* until very recently (Lvovsky 2015). After that, 14 species with various distributions were described by Meyrick, successively from 1906 to 1935, of which seven were placed directly in *Meleonoma*, viz. *M. heterota* Meyrick, 1914a and *M. petrota* Meyrick, 1914a, both published along with the generic description; *M. psammota* Meyrick, 1915; *M. implexa* Meyrick, 1918; *M. nephospora* Meyrick, 1930; *M. pardalias* Meyrick, 1931; *M. impulsa* Meyrick, 1934. The other seven were originally published in various genera, and transferred into *Meleonoma* later on, viz. *M. capnodyta* (Meyrick, 1906) in *Borkhausenia* Hübner, [1825] 1816; *M. crocomitra* (Meyrick, 1914b) in *Pseudodoxia* Durrant, 1895; *M. facunda* (Meyrick, 1910b) in *Leptosaces* Meyrick, 1888; *M. stomota* (Meyrick, 1910a), *M. aridula* (Meyrick, 1910c), *M. malacobyrsa* (Meyrick, 1921) and *M. torophanes* (Meyrick, 1935) in *Cryptolechia*, Zeller, 1852. Years later, Viette (1955) reported the new species *M. diehlella* from Madagascar. Li and Wang (2002, 2004) reported five new species from China. Lvovsky (2015) described five new species from Nepal and China and transferred *Cryptolechia peditata* Wang, 2006b into *Meleonoma*. Yin and Wang (2016a, b) reported two new species from Taiwan and four new ones and one new record from Thailand. In the same year, Park and Park (2016) described two new species and one new record from Korea.

*Meleonoma* are mostly small to medium-sized moths, mainly distributed in the Australian, Afrotropical, Palearctic, and Oriental faunal regions; the genus is especially rich in the Oriental faunal region. *Meleonoma* is characterized by the front of the head usually covered with appressed scales and the vertex with erect and hairlike scales; the labial palpus bearing three segments and recurved upwardly, extending well beyond the vertex, with the third segment shorter than the second one; the scape without pecten; the tibia of all legs clothed with long hairs above; the forewing with ground color usually yellow or black or approaching one of these two colors, forewing patterns diverse, usually with an oblique, wide, yellow or dark brown fascia. The venation of the forewing is as follows: R1 from about middle of cell, R4 and R5 arising from upper angle of cell and stalked at the half of their length, R5 reaching to near apex, M1 and M2 parallel, M2, M3 and CuA1 all arising from near the lower angle of the cell and separated from each other, CuA1 and CuA2 parallel, CuP weakly developed. The venation of hindwings is as follows: Rs, M1 and M2 nearly parallel, M3 and CuA1 stalked at the base or arising from the same point of the cell, CuA2 far from CuA1, arising from about 4/5 of the posterior margin of the cell, and CuP well developed. Tergum II-VII of abdomen with patches of a broad area of directed setae (Figs 3, 4). *Meleonoma* can also be identified by some key characters, such as the male genitalia with a well-developed uncus, a partly sclerotized circular or entirely membranous gnathos, and a well-defined saccus; by the female genitalia with an entirely or partly sclerotized ductus bursae, and one signum, often with spines if present (Wang 2006a; Yin and Wang 2016b; Kim and Lee 2017).

Nothing is known about their host plants.

The taxonomic status of the genus is controversial and the genus has been placed in different families and subfamilies of the Gelechioidea. It was originally described in the family Oecophoridae (Meyrick 1914a). After that, Clarke (1965) placed *Meleonoma* in the Cosmopterigidae (without any comments). Since then, many researchers followed his treatment (e.g., Nye and Fletcher 1991; Li and Wang 2002, 2004). Lvovsky (2015) transferred the genus into the Lypusidae. The most recent phylogenetic study of Kim et al. (2016) indicated that *Meleonoma* most likely belongs to the Xyloryctidae. Although the taxon sampling in Kim et al. (2016) was limited for this genus, their work is currently the only one based on molecular phylogenetic evidence. Therefore, we tentatively follow this and place *Meleonoma* in the Xyloryctidae.

In this study, two new species are described from China: *M. foliiformis* Yin, sp. nov. from Guangxi Province and *M. projecta* Yin, sp. nov. from Fujian Province. The species number of this genus is thus increased to 37.

## Material and methods

The examined specimens were collected from Guangxi and Fujian Provinces in southeastern China in 2018. The descriptive terminology of the anatomical structures generally follows Wang (2006a), however in descriptions of the male genitalia, the more proper term phallus rather than aedeagus is applied here following Kristensen (2003). Photographs of adults were taken using a Canon EOS 6D Mark II camera plus an EF 100 mm f/2.8L MACRO IS USM lens with the help of EOS Utility 3.10.20 software. Images of genitalia were captured using a Leica DM4 B upright microscope and photomontage was performed with Leica Application Suite X imaging software. All type specimens are deposited in the Morphological Laboratory, Guizhou University of traditional Chinese Medicine, Guiyang 550025, Guizhou, China.

## Taxonomy

### Genus *Meleonoma* Meyrick, 1914a

*Meleonoma* Meyrick, 1914a: 255. Type species: *Cryptolechia stomota* Meyrick, 1910a, by original designation.

=*Acryptolechia* Lvovsky, 2010: 378. Type species: *Cryptolechia malacobyrsa* Meyrick, 1921. Synonymised by Lvovsky (2015).

### Checklist of *Meleonoma* Meyrick in China

1 *Meleonoma apicispinata* Wang, 2016b: 26

Distribution: China (Taiwan Province).

2 *Meleonoma echinata* Li, 2004: 38

Distribution: China (Guizhou Province).

3 *Meleonoma facialis* Li et Wang, 2002: 230

Distribution: China (Henan, Jiangxi, Shaanxi, Sichuan, Yunnan Provinces), Indonesia, Korea, Nepal, Russia, Thailand.

4 *Meleonoma facunda* (Meyrick, 1910b): 155

Distribution: China (Northern and Eastern, Zhejiang Province), India, Japan.

5 *Meleonoma flavimaculata* (Christoph, 1882): 29

Distribution: China (Heilongjiang Province), Russia.

6 *Meleonoma foliata* Li, 2004: 37

Distribution: China (Guangdong Province).

7 *Meleonoma foliiformis* Yin, sp. nov.

Distribution: China (Guangxi Province).

8 *Meleonoma malacobyrsa* (Meyrick, 1921): 394

Distribution: China (Anhui, Fujian, Guizhou, Henan, Hunan, Jiangsu, Jiangxi, Shaanxi, Sichuan, Taiwan, Zhejiang Provinces), Japan, Korea.

9 *Meleonoma malacognatha* Li et Wang, 2002: 230

Distribution: China (Shaanxi Province).

10 *Meleonoma margisclerotica* Wang, 2016b: 25

Distribution: China (Taiwan Province).

11 *Meleonoma meyricki* Lvovsky, 2015: 773

Distribution: China (Yunnan Province).

12 *Meleonoma pardalias* Meyrick, 1931: 191

Distribution: China (Sichuan Province).

13 *Meleonoma peditata* (Wang, 2006b): 8

Distribution: China (Hubei Province).

14 *Meleonoma polychaeta* Li, 2004: 35

Distribution: China (Hunan Province).

15 *Meleonoma projecta* Yin, sp. nov.

Distribution: China (Fujian Province).

16 *Meleonoma torophanes* (Meyrick, 1935): 81

Distribution: China (Henan, Hubei, Shaanxi, Shanghai, Zhejiang Provinces), Korea.

#### Meleonoma foliiformis

Taxon classificationAnimaliaLepidopteraXyloryctidae

Yin sp. nov.

93068800-3119-5fb5-a7e4-8c1e2d8ca8f8

http://zoobank.org/0BC78FAD-DA43-4D1A-BD8D-80A0DE89EA1C

Figs 1 , 3 , 5

##### Material examined.

**Holotype**: China • ♂; Guangxi Province, Daming Mountain; alt. 1200 m, 4 Jun. 2018; Yuping Li leg.; YAH18108. **Paratype**: 1 ♂, same collection data as for preceding; YAH19001.

##### Diagnosis.

This new species resembles *M. facunda* (Meyrick, 1910b) in both appearance and genitalia. The differences between *M. foliiformis* and *M. facunda* in the male genitalia are as follows: *M. foliiformis* with the ventral process of the sacculus in a distinct leaf shape and the phallus with the distal 1/4 hooked; *M. facunda* with the ventral process of sacculus tiny, triangular in shape and the phallus straight.

##### Description.

Head: vertex mixed with pale gray scales, front pale yellow; labial palpus long and recurved, extending well beyond vertex, with smooth scales; outer surface of labial palpus with segment 1 and distal half as well as end of segment 2 densely covered with dark-brown scales, inner surface yellow; segment 3 yellow, about 3/4 length of segment 2; antenna with scape pale yellow; flagellum alternately pale yellow and dark brown on dorsal surface, ventral surface pale yellow; ocelli absent; scales of proboscis pale yellow.

**Figures 1–6.**
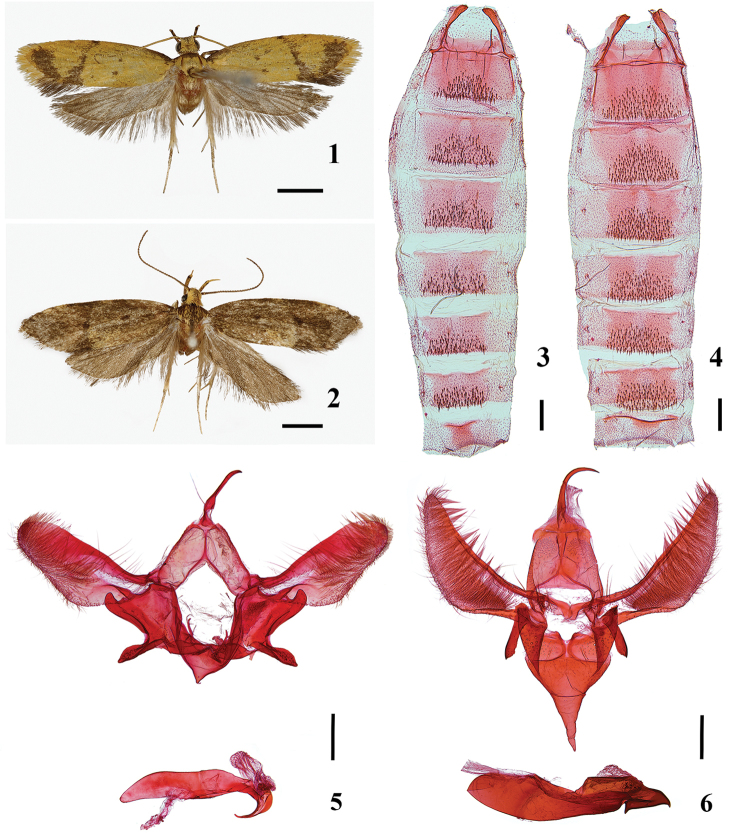
*Meleonoma* species, morphology **1** adult of *Meleonoma foliiformis* Yin, sp. nov., holotype, male (gen. slide no. YAH18108) **2** adult of *M. projecta* Yin, sp. nov., holotype, male (gen. slide no. YAH18125) **3** abdomen of *M. foliiformis* Yin, sp. nov., holotype, male (gen. slide no. YAH18108) **4** abdomen of *M. projecta* Yin, sp. nov., holotype, male (gen. slide no. YAH18125) **5** male genitalia of *M. foliiformis* Yin, sp. nov., paratype, phallus illustrated separately (gen. slide no. YAH19001) **6** male genitalia of *M. projecta* Yin, sp. nov., holotype, phallus illustrated separately (gen. slide no. YAH18125). Scale bar: 2.00 mm (**1, 2**); 0.25 mm (**3−6**).

Thorax: Tegula and mesonotum blackish brown anteriorly, yellow posteriorly; legs whitish yellow, tibiae scattered with blackish brown scales and tarsi with blackish brown speckles on outside. Forewing (Fig. 1): Length 6.0–7.0 mm (*N* = 2), about 3.5 X as long as wide, along costa with blackish brown streak from base to about basal 1/5, distal 1/3 of costa with several blackish brown dots; a dark-brown fascia extending from near middle of costa obliquely to tornus, slightly wider posteriorly; cell with 2 blackish brown dots, one set at middle of cell, the other set at distal 2/5 of fold; apex dark brown, somewhat forming a triangular patch along termen; cilia yellow except dark brown on tornus. Ventral surface yellowish brown. Hindwing (Fig. 1): translucent grayish brown, gradually darkening towards apex; cilia grayish brown.

Abdomen (Figs 3, 5): Male genitalia (Fig. 5): Uncus long and thin, wider basally, sparsely setose at basal 2/5; tegumen inverted V-shaped, lateral arms about same width, posterior margin arched inwards, anterior margin deeply concave, V-shaped; valva gradually widening to middle from a narrow base, distal half long oval in shape, distal half of ventral surface densely covered with long hairs; costa broadly arched forming a shallow notch; transtilla short and weakly sclerotized, with rounded apex; sacculus with basal 1/3 of dorsal margin joined with valva, two sclerotized processes at end of dorsal and ventral margin respectively: dorsal process somewhat semicircular, ventral process leaf-shaped, outer margin heavily sclerotized; saccus inverted triangular in shape, apex narrowly rounded; juxta U-shaped; phallus with basal 1/4 thin, gradually thickened to about 1/4, nearly same width from basal 1/4 to about distal 1/4, distal 1/4 hooked, apex pointed. Female genitalia: unknown.

##### Biology.

The host plant of the larva stage is unknown. The adults were collected using lamp attraction in June.

##### Distribution.

China (Guangxi Province).

##### Etymology.

The specific name, the Latin adjective *foliiformis*, means leaf-like, and refers to the leaf-shaped ventral process of the sacculus.

#### Meleonoma projecta

Taxon classificationAnimaliaLepidopteraXyloryctidae

Yin sp. nov.

df27b1b6-eacc-5f5d-b741-c08739d3283d

http://zoobank.org/EE0501C4-54A9-4B6D-A7A5-30EB59F0EA0C

Figs 2 , 4 , 6

##### Material examined.

**Holotype**: China • ♂, Fujian Province, Wuyi Mountain; alt. 1200 m, 10 May 2018; coll. Yuping Li leg.; YAH18125. **Paratype**: 1 ♂, same collection data as for preceding; YAH19002.

##### Diagnosis.

This new species can be distinguished from its congeners easily by the unique character in the male genitalia. In *M. projecta*, the distal portion of the sacculus with a heavily sclerotized process. It is also slightly similar to *M. malacobyrsa* externally. They can be distinguished by the fascia in the forewings: *M. projecta* with the fascia indistinct, whereas it is clearly outlined in *M. malacobyrsa*. In *M. projecta* the valva has no odontoid process on the ventral margin, sacculus with apex produced and phallus with tiny teeth near apex; *M. malacobyrsa* has the valva with an odontoid process on the ventral margin, sacculus concave and without a tooth on the phallus.

##### Description.

Head: vertex with grayish brown scales at middle, front yellow; labial palpus long and recurved, extending well beyond vertex, with smooth scales, outer surface of labial palpus with segment 1 and segment 2 dark brown, inner surface yellow, apex of segment 2 with blackish brown dots; segment 3 yellow, scattered with blackish brown dots, about 1/2 of second segment; antenna with scape earthy yellow on ventral surface and blackish brown on dorsal surface, with flagellum ringed, alternately blackish brown and earthy yellow; ocelli absent; scales of proboscis yellow.

Thorax: Tegula and mesonotum blackish brown mixed with yellow; legs light yellow, grayish brown on ventral surface, with grayish brown speckles on outside surface of tibiae and tarsi. Forewing (Fig. 2): Length 7.0–8.0 mm (*N* = 2), about 3.0 X longer than wide, blackish brown mixed with yellow scales; costa with a large diffused yellow blotch at about distal 1/5. Ventral surface dark brown; an oblique dark brown fascia running from basal 2/3 of costa to tornus; cell with 2 blackish brown markings, set at middle and end of cell respectively; cilia dark brown except yellow basally. Hindwing (Fig. 2): grayish brown; cilia grayish brown. Ventral surface of forewing and hindwing dark brown.

Abdomen (Figs 4, 6): Male genitalia (Fig. 6): Uncus with basal 1/4 triangular in shape, distal 3/4 long and hooked, pointed at apex; gnathos weakly sclerotized at base forming two elliptic sclerites, other parts membranous; tegumen inverted V-shaped, lateral arms gradually narrowed to apex, posterior margin straight, anterior margin deeply concave, onion-shaped; valva somewhat knife-shaped, gradually widening to basal 2/5 from a narrow base, distal 3/5 gradually tapered to rounded apex, ventral surface densely covered with long hairs; costa slightly arched inwardly; transtilla greatly protruded forward medially, distal portion curving downward and in contact with each other; sacculus jointed with valva dorsally; distal portion with a long heavily sclerotized process, bladelike, pointed at apex; saccus funnel-shaped, narrowly rounded at apex; phallus with basal 2/3 elongately ovate, distal 1/3 irregularly shaped, bearing three small teeth at distal 1/4 and end. Female genitalia: unknown.

##### Biology.

The host plant of the larva stage is unknown. The adults were collected using lamp attraction in May.

##### Distribution.

China (Fujian Province).

##### Etymology.

The specific name, the Latin adjective *projectus*, refers to the heavily sclerotized process of the sacculus.

## Supplementary Material

XML Treatment for Meleonoma foliiformis

XML Treatment for Meleonoma projecta
